# Prognostic relevance of LGALS3BP in human colorectal carcinoma

**DOI:** 10.1186/s12967-015-0606-x

**Published:** 2015-07-30

**Authors:** Enza Piccolo, Nicola Tinari, Domenica D’Addario, Cosmo Rossi, Valentina Iacobelli, Rossana La Sorda, Rossano Lattanzio, Maurizia D’Egidio, Annalisa Di Risio, Mauro Piantelli, Pier Giorgio Natali, Stefano Iacobelli

**Affiliations:** MediaPharma s.r.l., Via dei Vestini, 31, Chieti, Italy; Department of Experimental and Clinical Sciences, “G. D’Annunzio” University and Foundation, Chieti, Italy; Department of Ob/Gyn, University la Sapienza, Rome, Italy; Regina Elena Cancer Institute, Rome, Italy

**Keywords:** LGALS3BP, β-Catenin, Colorectal cancer, Prognosis

## Abstract

**Background:**

A previous report has shown that LGALS3BP (also known as 90K or Mac-2 BP) has antitumor activity in colorectal cancer (CRC) via suppression of Wnt signalling with a novel mechanism of ISGylation-dependent ubiquitination of β-catenin. The role of LGALS3BP in CRC prognosis was investigated.

**Methods:**

The role of LGALS3BP on CRC progression and clinical prognosis was analyzed by combining cell cultures, in vitro assays, and immunohistochemistry.

**Results:**

Silencing of LGALS3BP in HCT-116 human colon cancer cells resulted in enhanced β-catenin expression that was reversed by addition of human recombinant LGALS3BP. Moreover, intra-tumor delivery of LGALS3BP reduced tumor growth of xenografts originating from LGALS3BP-silenced HCT-116 cells. Finally, in a series of 196 CRC patients, LGALS3BP expression in tumor tissue associated with clinical outcome. Patients with high LGALS3BP expression had lower risk of relapse and a longer overall survival time than those with low LGALS3BP expression. Multivariate analyses confirmed LGALS3BP expression status as the only independent prognostic factor of survival.

**Conclusions:**

These results provide evidence that low expression of LGALS3BP participates in malignant progression of CRC and implicates poor prognosis, highlighting its augmentation as a potential therapeutic approach.

## Background

Colorectal cancer is a major cause of cancer-related mortality worldwide, causing ~500,000 deaths annually. Following curative resection, there is a considerable risk of recurrence in patients with stage II and III disease. Recurrence occurs in ~20% of stage II patients and ~50% of the stage III patients may be cured with surgery alone [1–3]. Therefore, it is critical to identify patients with a high risk of recurrence.

LGALS3BP is a large oligomeric, highly glycosylated protein composed of ≈90 kDa subunits that was originally identified as a tumor-secreted antigen [4] and as a ligand of the lactose-specific S-type lectin, galectin-3 (formerly Mac-2) [5]. Whereas the bio-physiological activity of LGALS3BP is not yet well defined, accumulating evidence has shown that the protein may be involved in cancer growth and progression [6]. Notably, significantly elevated expression of LGALS3BP in the serum or tumor tissue has been found to be associated with poor clinical outcome in patients with breast carcinoma [7, 8], hepatocellular carcinoma [9, 10], pleural mesothelioma [11], pancreatic carcinoma [12], non-small cell lung carcinoma [13] and neuroblastoma [14]. In contrast, positive effects of LGALS3BP on cancer prognosis have also been reported [15, 16]. Recently, Lee et al. [16] found that LGALS3BP has antitumor activity in colorectal cancer (CRC) cells via suppression of Wnt signalling with a novel mechanism of ISGylation dependent ubiquitination of β-catenin. The authors also found that LGALS3BP knockdown resulted in increased tumor growth and metastasis formation in a syngeneic mouse colon tumor model. In the present study, we confirm and extend these findings by showing that LGALS3BP knock-down human CRC cells formed large tumors when implanted in nude mice and that intra-tumor delivery of human recombinant LGALS3BP induced regression of established CRC xenografts. In addition, we show that high LGALS3BP expression in the tumor tissue is associated with a longer disease-free and overall survival in CRC patients.

## Methods

### Cell lines and culture

The HCT-116 human colon cancer cell line was obtained from ATCC (Rockville, MD, USA). Cells were maintained in culture for fewer than 6 months after thawing. Cells were maintained in RPMI-1640 medium (Invitrogen, Carlsbad, CA. USA) with 10% heat-inactivated fetal bovine serum (FBS; Invitrogen), l-glutamine and antibiotics (Sigma Aldrich Corporation, St. Louis, MO, USA). The cells were maintained in a humidified chamber with 95% air and 5% CO_2_ at 37°C.

### LGALS3BP gene knockdown

A 21-nucleotide sequence corresponding to nucleotide 2216–2236 of human LGALS3BP mRNA (NCBI Accession NM-005567.3) or a 21-nucleotide sequence with no significant homology to any mammalian gene sequence serving as a non-silencing control (OligoEngine, Hercules, CA, USA) were inserted into the pSUPER.retro.puro (OligoEngine). After transformation of DH5α competent cells (Invitrogen), the recombinant plasmids were confirmed by PCR amplification, restriction enzymes digestion and DNA sequencing.

The generation of HCT-116 knock-down cells was performed according to the methods described in our previous report [17].

### Enzyme-linked immunosorbent assay (ELISA)

A sandwich-type ELISA (Diesse, Siena, Italy) was used to determine the concentration of LGALS3BP in the conditioned medium of control- and LGALS3BP-knock-down HCT-116 cells. Culture medium was used a blank control.

### Generation of recombinant LGALS3BP

Human recombinant LGALS3BP was immunoaffinity-purified [18] from serum-free supernatant of human embryonic kidney EBNA-293 cells (Invitrogen) transfected with LGALS3BP cDNA [19]. In brief, the supernatant of the cells (2 L) added with Pefabloc (Boehringer Mannheim, Germany) and EDTA (1 and 0.4 mM, respectively), was concentrated with a Vivaflow 200 system (Sartorius Biotech Goettingen, Germany) to 50 mL and passed over an affinity column made of 20 mg of the anti-LGALS3BP antibody (SP-2) covalently coupled to 12 mL of cyanogen bromide activated Sepharose CL-4B (Sigma Aldrich Corporation). After washing the column with PBS, bound proteins (>95% LGALS3BP) were eluted with 20 mL of 0.1 M glycine buffer, pH 2.8. Pooled LGALS3BP-containing fractions were dialysed against PBS and stored in small aliquots at −80°C. SDS-PAGE showed a major band (90%) migrating at ~97 kDa. The endotoxin level of the final preparation was <5 EU/μg, as evaluated by the Lymulus Amebocyte Lysate (LAL) test (Clongen Labs, Germantown, MD, USA).

### Confocal microscopy

HCT-116shctrl and HCT-116shLGALS3BP were seeded on glass coverslips and allowed to grow for 24 h at 37°C in 5% CO_2_. Cells were incubated with LGALS3BP (10 μg/mL) for the indicated times, fixed with 4% paraformaldehyde for 15 min at room temperature, permeabilized with 0.25% Triton X-100 for 5 min and blocked with 0.1% bovine serum albumin for 1 h at room temperature. Coverslips were then incubated for 2 h at room temperature with a mouse anti β-catenin antibody (clone 14/β-catenin, Becton–Dickinson, Franklin Lakes, NJ, USA) followed by Alexa-Fluor 488 goat anti-mouse secondary antibody (Molecular Probes, Life Technologies, Paisley, UK). DRAQ5 (Vinci Biochem, Firenze, Italy) was used to visualize nuclei. Images were acquired with a Zeiss LSM 510 meta-confocal microscope (Zeiss, Oberkochen, Germany) using 488 and 633 nm lasers. Detector gain voltages and pinhole were set at the beginning of the experiment and maintained constant during the acquisition of all samples.

### Western blotting

Cells were lysed with RIPA buffer containing protease and phosphatase inhibitors (Sigma Aldrich Corporation). Lysates were clarified by centrifugation at 14,000× rpm for 15 min at 4°C, subjected to 10% SDS-PAGE and Western blotting using a mouse anti-β-catenin antibody (Becton–Dickinson), a mouse anti-actin antibody (Sigma Aldrich Corporation) or a mouse monoclonal antibody against LGALS3BP (3C12.2). Incubation was performed overnight at 4°C. After washing with PBS containing 0.1% Tween-20, blots were incubated with a goat anti-mouse HRP-conjugated IgG as a secondary antibody (Biorad, Berkeley, CA, USA) at room temperature for 2 h and developed with a chemiluminescence detection system (Perkin-Elmer, Waltham, MA, USA).

### Tumor xenografts

All animal studies were approved by the Institutional Animal Ethics Committee. Female athymic (nu+/nu+) mice (6-week old) (Charles River Laboratories, Milan, Italy) were acclimatized for 2 weeks before the start of the experiments and housed under specific pathogen-free conditions. Mice were given ad libitum access to food and water. HCT-116shLGALS3BP or HCT-116shctrl cells (5 × 10^6^) were implanted s.c. into the right flank of the mice (15 mice for HCT-116shLGALS3BP cells; 9 mice for HCT-116shctrl cells). Tumor volume was monitored twice a week for a total of 6 weeks by a caliper and calculated using the following formula: tumor volume (mm^3^) = (length × width^2^)/2. In another set of experiments, animals harboring HCT-116shLGALS3BP xenografts (approximately 200 mm^3^) were randomly divided into two groups of 10 animals each; one group was injected intra-tumorally with 50 μL LGALS3BP (100 μg), while the other group was injected with the same volume of PBS. Injections were made twice a week. Animals received a total of nine injections.

### Patient information and tissue specimens

A total of 196 assessable CRCs were collected from patients who received surgical treatment at the University “G. D’Annunzio”, Chieti, Italy between 1996 and 2010. Inclusion criteria were: (a) CRC primary cancer; (b) CRC with pathological diagnosis; (c) informed consent or waiver of consent; (d) age ≥18 years; (e) receipt of at least one follow-up within 5 years. To avoid possible interactions between response to treatment and LGALS3BP status, only patients not receiving any adjuvant systemic therapy were included in the study. The clinico-pathological classification and the stage were determined according to the American Joint Committee on Cancer (AJCC) TNM staging system. Each lesion was graded histologically according to the WHO classification criteria. Patients and tumor characteristics are summarized in Table 1. The median follow-up was of 45 months (range 1–176 months). During follow-up, 63 out of 196 (32%) patients developed relapses and deaths were observed in 50 out of 196 (26%) patients. The study was reviewed and approved by Institutional Research Ethics Committee and written informed consent was obtained from all patients.

**Table 1:** Clinico-pathological data of 196 patients with CRC

	Number of cases (%)
Gender	
Male	125 (64)
Female	71 (36)
Age (years)	
Median	71
Range	31–89
Location	
Colon	161 (82)
Rectal	35 (18)
Clinical stage	
1	14 (7)
2	155 (80)
3	25 (13)
	TOT 194
Pathological differentiation	
Well	16 (8)
Moderate	163 (84)
Poor	16 (8)
	TOT 195
LGALS3BP	
Low	151 (77)
High	45 (23)

### Immunohistochemistry

For the evaluation of β-catenin expression in mouse xenografts, formalin-fixed and paraffin-embedded tumor xenografts of HCT-116shctrl (n = 9) and HCT-116shLGALS3BP (n = 15), were sectioned at 5 μm and stained using anti-human β-catenin mouse monoclonal antibody (BD Transduction Laboratories) at 1:3,000 dilution for 60 min. Antigen retrieval was performed by microwave treatment at 750 W for 10 min in 10 mmol/L sodium citrate buffer (pH 6.0). EnVision kit (K4001, Dako, Glostrup, Denmark) was used for signal amplification. In control sections the specific primary antibody was replaced with isotype-matched immunoglobulins (Dako).

Tissue microarrays (TMA) were constructed by extracting 2-mm diameter cores of histologically confirmed neoplastic areas from 196 invasive primary human CRC, as previously detailed [20] TMA sections were stained using the monoclonal mouse anti-human LGALS3BP as previously reported [8]. Staining of LGALS3BP was quantified as percentage of stained tumor cells. To dichotomize LGALS3BP expression, a cut-off value of 69% was chosen, which corresponded to the 75th percentile. Therefore, tumors whose percentage of stained cells was ≤69% were considered as low LGALS3BP, all the others as high LGALS3BP. Immunohistochemical analysis was done by two pathologists (MP, RL) who were blinded to the clinical data of the patients.

### Statistical methods

Two-tailed unpaired T-test was used to compare the statistical significance of the differences in data from two groups, where appropriate. Disease-free survival (DFS) was defined as the time from surgery to the first one of the following events: recurrence at local or distant sites, or intercurrent death without recurrence. Overall survival (OS) was defined as the interval between the date of surgery and date of death or the last known follow up. Survival curves were plotted by the Kaplan–Meier method and compared using the log-rank test. The association of LGALS3BP expression with outcome, adjusted for other prognostic factors, was tested by Cox’s proportional hazards model. The following covariates were included in the multivariate models: gender, tumor location, grade and LGALS3BP status. All statistical analyses were performed using by the SPSS 15.0 statistical software package (SPSS Inc., Chicago, IL, USA); p < 0.05 was considered as statistically significant.

## Results

### LGALS3BP-silenced CRC cells grow larger tumors, an effect which is reversed by intratumor injection of LGALS£BP

**Fig. 1:**
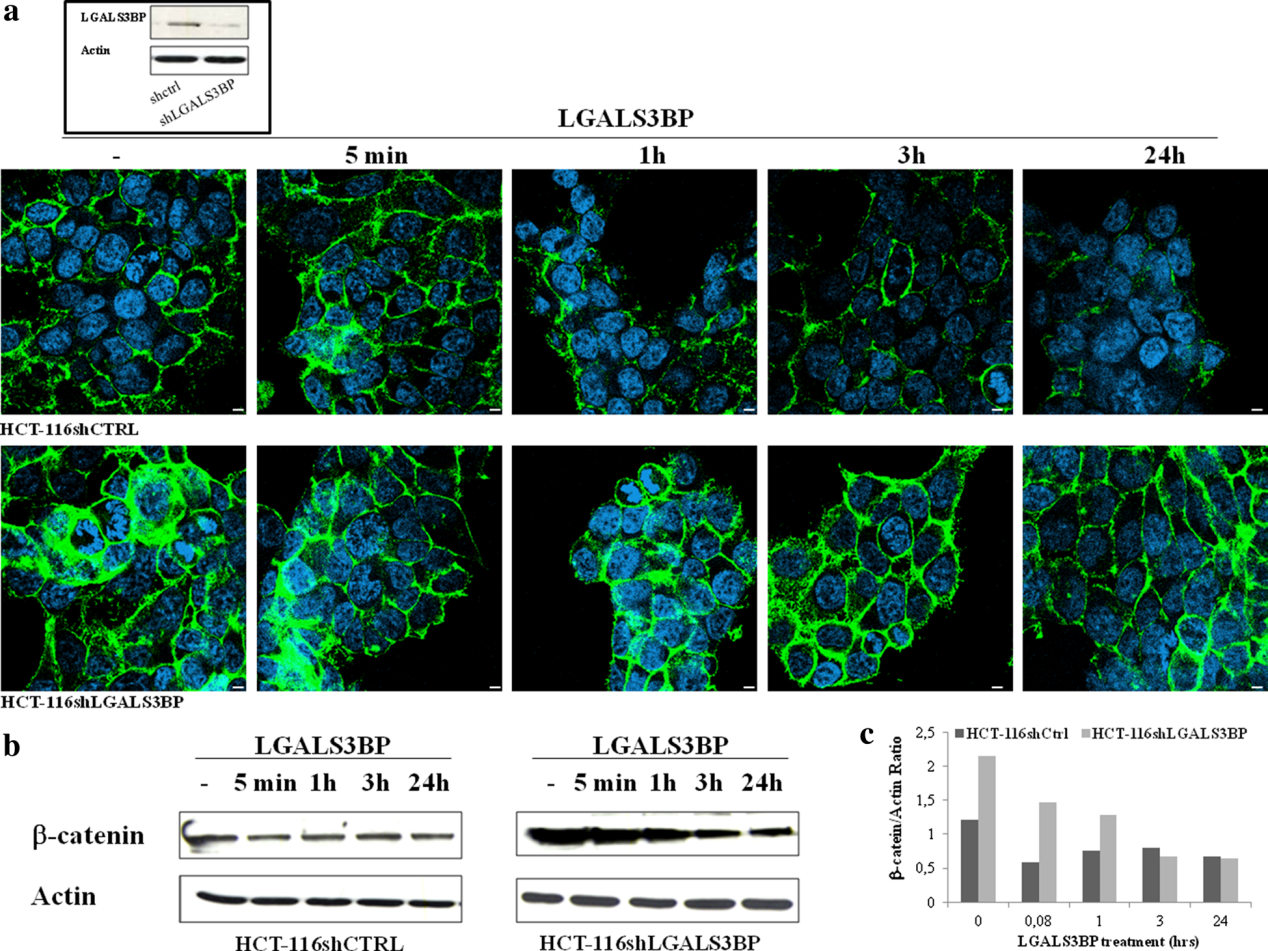
LGALS3BP downregulates β-catenin expression in human colon cancer cells. Expression of β‐catenin in HCT‐116shCTRL and HCT‐116shLGALS3BP HCT‐116 cells after stimulation with LGALS3BP (10 μg/mL) for the indicated times. **a** Confocal microscopy images showing β-catenin (*green*) and nuclei (*blue*) (*scale bar* 20 μm). In the *box*, western blotting analysis showing LGALS3BP expression in shCtrl and shLGALS3BP cells. **b** Representative western blots showing β-catenin expression. Actin was used as an internal loading control. **c** Histograms depict the band intensity ratio β-catenin/actin as measured by Image J software. Data are representative of at least three independent experiments.

To investigate the role of LGALS3BP on tumor growth, short hairpin RNA constructs were generated to stably knock-down LGALS3BP in HCT-116 cells (HCT-116shLGALS3BP). A scramble siRNA was also transfected into cells as negative control (HCT116shctrl). After transfection, expression of LGALS3BP protein was assessed by Western blotting (Fig. 1 box) and ELISA assay on the conditioned medium of both cell lines (185.5 ng/mL in HCT-116shctrl vs. 40.1 ng/mL in HCT-116shLGALS3BP cells).

As expected from the results of a previous report showing degradation of β-catenin after forced expression of LGALS3BP [16], stable knock-down of LGALS3BP led to a marked increase of β-catenin, as evaluated by confocal microscopy (Fig. 1a) and western blot analysis (Fig. 1b). Moreover, exposure of silenced cells to 10 μg/mL LGALS3BP resulted in a significant decrease of β-catenin expression both in HCT-116shctrl and HCT-116shLGALS3BP cells. After 24 h, the expression level of β-catenin in silenced cells was similar to the basal level of vector control cells.

**Fig. 2:**
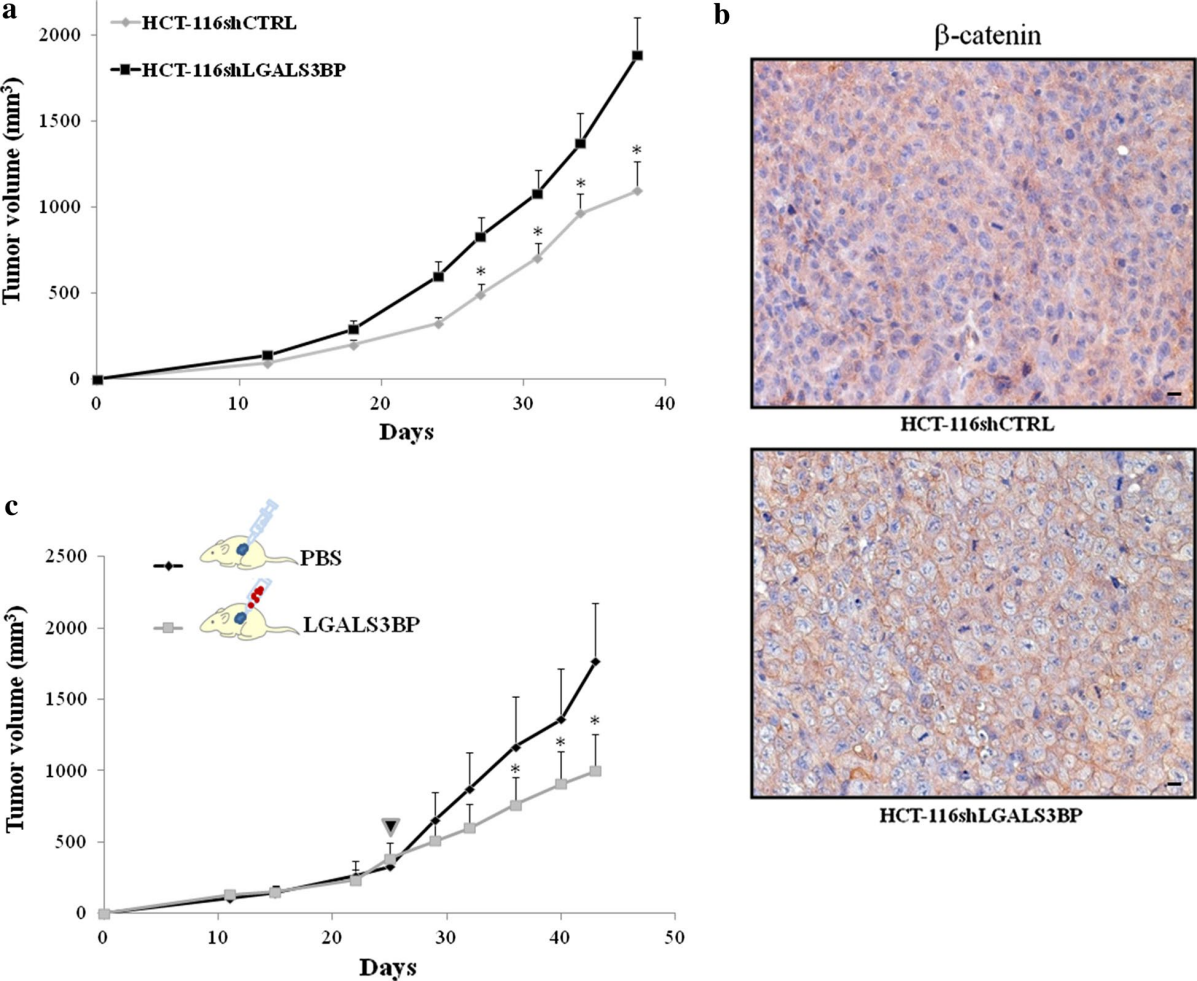
LGALS3BP knock-down affects growth of tumor xenografts and is associated with a reduction in β-catenin expression. **a** HCT-116shctrl and HCT-116shLGALS3BP cells were implanted s.c. into the right flank of female nude mice (9 mice for HCT-116shctrl; 15 mice for HCT-116shLGALS3BP). Tumor growth was assessed as described in “Methods”. *p < 0.05. **b** Examples of immunohistochemical staining of β-catenin in HCT-116shctrl and HCT-116shLGALS3BP xenografts (*scale bar* 20 μm). **c** Animals harboring HCT-116shLGALS3BP xenografts (approximately 200 mm^3^) were randomly divided into two groups (indicated by the *arrow*); tumors from one group were injected intra-lesionally with 100 μg LGALS3BP in 50 μL PBS, the other injected with the same volume of PBS. Injections were made twice a week. Tumor growth was assessed as described in “Methods”. (*p < 0.05).

To examine the function of LGALS3BP in tumorigenesis in vivo, we implanted s.c. HCT-116 vector control and LGALS3BP knock-down cells into the flanks of nude mice and monitored tumor growth for up to 6 weeks. A significant reduction in growth was seen in tumors deriving from vector control cells as compared to those deriving from LGALS3BP knock-down cells (58% reduction of control group compared to LGALS3BP knock-down group; p < 0.05; Fig. 2a). To investigate whether the higher growth rate of LGALS3BP knock-down tumors was linked to increased β-catenin expression, we performed immunohistochemistry. When compared to tumors deriving from HCT-116 vector control, those from LGALS3BP knock-down cells displayed higher expression of β-catenin, preferentially at plasma membrane level. By independent-sample t-test, β-catenin was found to be more expressed in LGALS3BP knock-down HCT-116 cells (24.1% ± 5.3 SE; mean percentage ± standard error) compared to tumors deriving from HCT-116 vector control (9.8% ± 4.7 SE), with a trend toward statistical significance (*p* = 0.079). Examples of β-catenin staining, preferentially at plasma membrane level, of tumors deriving from vector control and LGALS3BP-knock down cells are illustrated in Fig. 2b. Overall, these results suggest that the increased tumorigenic hallmarks of HCT-116 cells following LGALS3BP silencing was linked to an increase expression of β-catenin, both in vitro and in vivo.

To confirm that LGALS3BP has a suppressive role in CRC growth, tumors from HCT-116 LGALS3BP knock-down cells were injected intra-lesionally with 100 μg LGALS3PBP in 50 μL PBS or the same volume of PBS (as a control) twice a week; starting from the fifth injection of LGALS3BP, a significant reduction in tumor growth was observed (Fig. 2c).

### Correlation of LGALS3BP expression with patient outcome

**Fig. 3:**
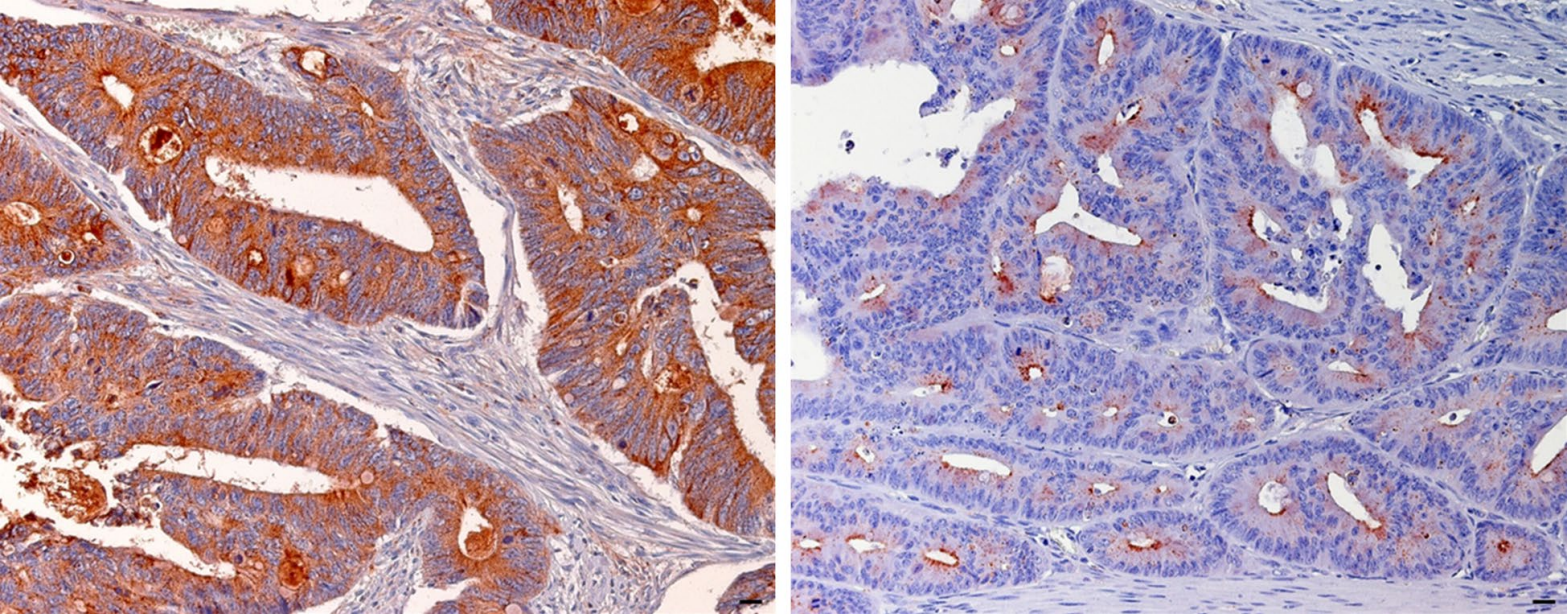
Examples of LGALS3BP staining in CRC. Immunohistochemical staining showing high (*left*) and low (*right*) expression of LGALS3BP in a case of CRC (original magnification ×400). *Scale bar* 20 μm.

LGALS3BP protein expression was evaluated by immunohistochemistry in 196 paraffin-embedded, archival primary colorectal cancer tissues. According to the cut-off, low LGALS3BP expression (staining of ≤69% of the neoplastic cells) was detected in 151/196 (77%) CRC tumors, while high LGALS3BP expression was detected in 45/196 (23%). LGALS3BP staining was cytoplasmic with diffuse and granular patterns and substantially confined to the neoplastic compartment. Examples of low and high expression of LGALS3BP are shown in Fig. 3. Low and high LGALS3BP expressing tumors did not differ significantly for the distribution of clinic-pathological variables evaluated (data not shown).

**Fig. 4:**
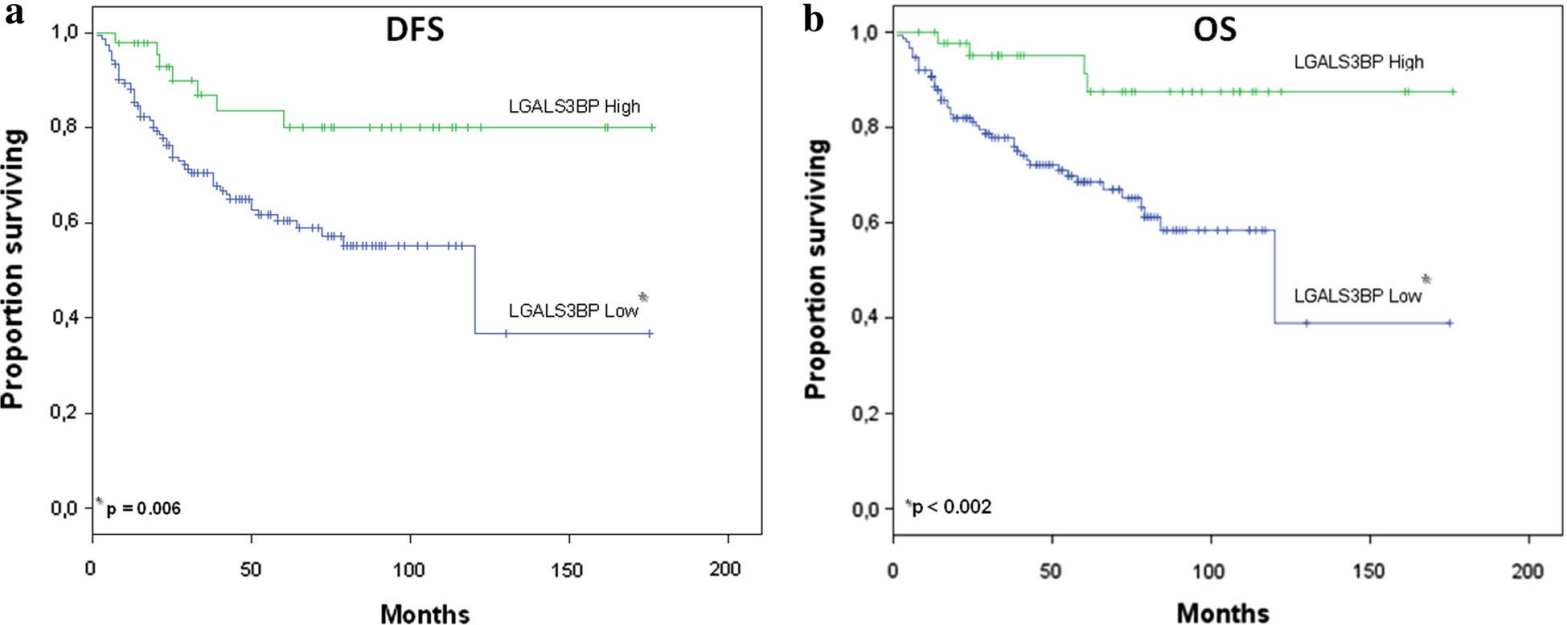
Correlation of LGALS3BP expression with patient outcome. Kaplan–Meier disease free survival (**a**) and overall survival (**b**) analysis among 196 CRC patients according to the expression of LGALS3BP in tumor tissue (p < 0.006 and p < 0.002, respectively). *Green* and *blue lines* indicate high and low expression of LGALS3BP, respectively.

Eight out of 45 (17.8%) patients with high LGALS3BP expressing tumors and 55 out of 151 (36.4%) patients with low LGALS3BP expressing tumors had a disease relapse. Analysis of Kaplan–Meier curves showed that patients with high LGALS3BP expressing tumors had a higher DFS rate than patients with low LGALS3BP expressing tumors (Fig. 4a). Multivariate analysis adjusted for the other prognostic factors showed that LGALS3BP status was the only significant prognostic parameter of DFS (HR 2 80, 95% CI 1.27–6.18; p = 0.011) (Table 2).

**Table 2:** Multivariate analysis of various prognostic parameters in patients with colorectal cancer

	Hazard ratio	95% CI	P
Disease free survival			
Gender (female vs. male)	1.06	0.62–1.79	0.84
Location (rectum vs. colon)	1.49	0.82–2.73	0.19
Tumor grade (2–3 vs. 1)	1.73	0.54–5.54	0.36
LGALS3BP (low vs. high)	2.80	1.27–6.18	0.011
Overall survival			
Gender (female vs. male)	1.11	0.61–2.03	0.74
Location (rectum vs. colon)	1.52	0.77–3.02	0.23
Tumor grade (2–3 vs. 1)	1.84	0.44–7.61	0.40
LGALS3BP (low vs. high)	4.07	1.45–11.45	0.008

Cox-regression analysis.

Patients whose tumors expressed low LGALS3BP had a shorter OS than those with high LGALS3BP expression (median OS 135 months vs. not reached, respectively; p < 0.002; Fig. 4b). The overall five-year cumulative survival rate was 68.5% in cases with low LGALS3BP expression and 91% in cases with high LGALS3BP expression. Furthermore, multivariate analyses indicated that LGALS3BP expression was the only significant prognostic factor of OS (HR 4.07, 95% CI 1.45–11.45; p = 0.008) (Table 2).

## Discussion

This is the first study on the prognostic relevance of the LGALS3BP in CRC patients. We demonstrated that high LGALS3BP expression in primary tumor tissue correlated with a better disease-free and overall survival outcome, whereas low LGALS3BP expression correlated with a poorer survival outcome. On multivariate analysis, LGALS3BP expression was an independent prognostic factor, suggesting that the protein may be a prognostic factor for survival in CRC patients. Since none of the patients received adjuvant systemic therapy, possible interactions between response to treatment and LGALS3BP status can be excluded, and the marker influence on survival can be attributed exclusively to its relationship with the natural history of the disease.

The role of LGALS3BP in cancer prognosis remains equivocal. The protein has been reported to have both negative and positive influences on the prognosis of various cancers. Most of the studies have shown that high LGALS3BP levels are associated with shorter survival, the occurrence of metastasis or a reduced response to chemotherapy [7, 11, 13, 21–24]. In contrast, positive effects of LGALS3BP have also been found. For example, engineered enhancement of LGALS3BP expression resulted in significant tumor growth inhibition [25] and high levels of LGALS3BP expression in tumor tissue were associated with a favorable outcome in a series of patients with Ewing’s sarcoma [15]. The mechanism underlying positive and negative influences of LGALS3BP on the prognosis of various cancers is not understood, but may be related to the multi-domain nature of the protein and its ability to bind to different ligands, including galectins, in particular galectin-3 and 1 [26], endosialin [27] and tetraspanins [28] in different tumor tissues.

Lee et al. [16] recently reported LGALS3BP-dependent suppression of Wnt signalling with a novel mechanism of ISGylation-dependent ubiquitination of β-catenin when it interacts with the tetraspanins CD9 and CD82. The authors examined the expression patterns of LGALS3BP, CD9, CD82, β-catenin and galectins in serial colon tissue sections in patients with stage I and IV CRC and also in metastatic liver tissues, and found that LGALS3BP, CD9 and CD82 were higher in the cancer tissues from stage I than in the stage IV and in the adjacent normal hepatic tissues than in the invading colon cancer cells. They speculated that a lower expression of LGALS3BP as well as CD9/CD82 in CRC tissues is a marker of poor prognosis of CRC.

Our results suggest that LGALS3BP reduction of β-catenin levels could represent a mechanism underlying LGALS3BP prognostic significance in CRC. Indeed LGALS3BP-silenced HCTI16 cells showed higher β-catenin levels as compared to control silenced cells (Fig. 1a, b) and developed larger tumors when injected into nude mice (Fig. 2a). The role of LGALS3BP as a suppressor of tumor growth was further substantiated by the finding that a significant tumor regression could be achieved with LGALS3BP injected directly into xenografts originating from LGALS3BP-silenced HCTI16 cells (Fig. 2c).

## Conclusion

In sum, our results suggest that a reduced expression of LGALS3BP is one of the factor responsible of the malignant progression of CRC and implicates poor prognosis This notion could represent a potential strategy for prevention or treatment of CRC growth and progression.

## Abbreviations

LGALS3BP: lectin, galactoside-binding, soluble, 3 binding protein
CRC: colorectal cancer
RPMI-1640: Roswell Park Memorial Institute
FBS: foetal bovine serum
ELISA: enzyme-linked immunosorbent assay
EDTA: ethylenediaminetetraacetic acid
RIPA: radioimmunoprecipitation assay
PBS: phosphate buffered saline
HRP: horse radish peroxidase
TMA: tissue microarray

## References

[1] Andre T, Quinaux E, Louvet C, Colin P, Gamelin E, Bouche O (2007). Phase III study comparing a semimonthly with a monthly regimen of fluorouracil and leucovorin as adjuvant treatment for stage II and III colon cancer patients: final results of GERCOR C96.1. J Clin Oncol.

[2] Gill S, Loprinzi CL, Sargent DJ, Thome SD, Alberts SR, Haller DG (2004). Pooled analysis of fluorouracil-based adjuvant therapy for stage II and III colon cancer: who benefits and by how much?. J Clin Oncol.

[3] Mamounas E, Wieand S, Wolmark N, Bear HD, Atkins JN, Song K (1999). Comparative efficacy of adjuvant chemotherapy in patients with Dukes’ B versus Dukes’ C colon cancer: results from four National Surgical Adjuvant Breast and Bowel Project adjuvant studies (C-01, C-02, C-03, and C-04). J Clin Oncol.

[4] Iacobelli S, Arno E, D’Orazio A, Coletti G (1986). Detection of antigens recognized by a novel monoclonal antibody in tissue and serum from patients with breast cancer. Cancer Res.

[5] Koths K, Taylor E, Halenbeck R, Casipit C, Wang A (1993). Cloning and characterization of a human Mac-2-binding protein, a new member of the superfamily defined by the macrophage scavenger receptor cysteine-rich domain. J Biol Chem.

[6] Grassadonia A, Tinari N, Iurisci I, Piccolo E, Cumashi A, Innominato P (2004). 90K (Mac-2 BP) and galectins in tumor progression and metastasis. Glycoconj J.

[7] Iacobelli S, Sismondi P, Giai M, D’Egidio M, Tinari N, Amatetti C (1994). Prognostic value of a novel circulating serum 90K antigen in breast cancer. Br J Cancer.

[8] Tinari N, Lattanzio R, Querzoli P, Natoli C, Grassadonia A, Alberti S (2009). High expression of 90K (Mac-2 BP) is associated with poor survival in node-negative breast cancer patients not receiving adjuvant systemic therapies. Int J Cancer.

[9] Correale M, Giannuzzi V, Iacovazzi PA, Valenza MA, Lanzillotta S, Abbate I (1999). Serum 90K/MAC-2BP glycoprotein levels in hepatocellular carcinoma and cirrhosis. Anticancer Res.

[10] Iacovazzi PA, Notarnicola M, Caruso MG, Guerra V, Frisullo S, Altomare DF (2010). Serum levels of galectin-3 and its ligand 90k/mac-2 bp in colorectal cancer patients. Immunopharmacol Immunotoxicol.

[11] Strizzi L, Muraro R, Vianale G, Natoli C, Talone L, Catalano A (2002). Expression of glycoprotein 90K in human malignant pleural mesothelioma: correlation with patient survival. J Pathol.

[12] Kunzli BM, Berberat PO, Zhu ZW, Martignoni M, Kleeff J, Tempia-Caliera AA (2002). Influences of the lysosomal associated membrane proteins (Lamp-1, Lamp-2) and Mac-2 binding protein (Mac-2-BP) on the prognosis of pancreatic carcinoma. Cancer.

[13] Marchetti A, Tinari N, Buttitta F, Chella A, Angeletti CA, Sacco R (2002). Expression of 90K (Mac-2 BP) correlates with distant metastasis and predicts survival in stage I non-small cell lung cancer patients. Cancer Res.

[14] Morandi F, Corrias MV, Levreri I, Scaruffi P, Raffaghello L, Carlini B (2011). Serum levels of cytoplasmic melanoma-associated antigen at diagnosis may predict clinical relapse in neuroblastoma patients. Cancer Immunol Immunother.

[15] Zambelli D, Zuntini M, Nardi F, Manara MC, Serra M, Landuzzi L (2010). Biological indicators of prognosis in Ewing’s sarcoma: an emerging role for lectin galactoside-binding soluble 3 binding protein (LGALS3BP). Int J Cancer.

[16] Lee JH, Bae JA, Lee JH, Seo YW, Kho DH, Sun EG (2010). Glycoprotein 90K, downregulated in advanced colorectal cancer tissues, interacts with CD9/CD82 and suppresses the Wnt/beta-catenin signal via ISGylation of beta-catenin. Gut.

[17] Piccolo E, Tinari N, Semeraro D, Traini S, Fichera I, Cumashi A (2013). LGALS3BP, lectin galactoside-binding soluble 3 binding protein, induces vascular endothelial growth factor in human breast cancer cells and promotes angiogenesis. J Mol Med (Berl).

[18] Silvestri B, Calderazzo F, Coppola V, Rosato A, Iacobelli S, Natoli C (1998). Differential effect on TCR:CD3 stimulation of a 90-kD glycoprotein (gp90/Mac-2BP), a member of the scavenger receptor cysteine-rich domain protein family. Clin Exp Immunol.

[19] Sasaki T, Brakebusch C, Engel J, Timpl R (1998). Mac-2 binding protein is a cell-adhesive protein of the extracellular matrix which self-assembles into ring-like structures and binds beta1 integrins, collagens and fibronectin. EMBO J.

[20] Lattanzio R, Marchisio M, La Sorda R, Tinari N, Falasca M, Alberti S (2013). Overexpression of activated phospholipase Cgamma1 is a risk factor for distant metastases in T1-T2, N0 breast cancer patients undergoing adjuvant chemotherapy. Int J Cancer.

[21] Iacovazzi PA, Guerra V, Elba S, Sportelli F, Manghisi OG, Correale M (2003). Are 90K/MAC-2BP serum levels correlated with poor prognosis in HCC patients? Preliminary results. Int J Biol Markers.

[22] Fornarini B, D’Ambrosio C, Natoli C, Tinari N, Silingardi V, Iacobelli S (2000). Adhesion to 90K (Mac-2 BP) as a mechanism for lymphoma drug resistance in vivo. Blood.

[23] Zhang DS, Ding Y, Li YH, Xu RH, Wang B, Zhang XS (2005). Expression of glycoprotein 90K in non-Hodgkin’s lymphoma and its clinical significance. Ai Zheng.

[24] Gentiloni N, Caradonna P, Costamagna G, D’Ostilio N, Perri V, Mutignani M (1995). Pancreatic juice 90K and serum CA 19-9 combined determination can discriminate between pancreatic cancer and chronic pancreatitis. Am J Gastroenterol.

[25] Jallal B, Powell J, Zachwieja J, Brakebusch C, Germain L, Jacobs J (1995). Suppression of tumor growth in vivo by local and systemic 90K level increase. Cancer Res.

[26] Lahm H, Andre S, Hoeflich A, Kaltner H, Siebert HC, Sordat B (2004). Tumor galectinology: insights into the complex network of a family of endogenous lectins. Glycoconj J.

[27] Becker R, Lenter MC, Vollkommer T, Boos AM, Pfaff D, Augustin HG (2008). Tumor stroma marker endosialin (Tem1) is a binding partner of metastasis-related protein Mac-2 BP/90K. FASEB J.

[28] Lee JH, Cho ES, Kim MY, Seo YW, Kho DH, Chung IJ (2005). Suppression of progression and metastasis of established colon tumors in mice by intravenous delivery of short interfering RNA targeting KITENIN, a metastasis-enhancing protein. Cancer Res.

